# Utilizing the Hippo pathway as a therapeutic target for combating endocrine-resistant breast cancer

**DOI:** 10.1186/s12935-021-01999-5

**Published:** 2021-06-10

**Authors:** Jing Chen, Runlan Wan, Qinqin Li, Zhenghuan Rao, Yanlin Wang, Lei Zhang, Alexander Tobias Teichmann

**Affiliations:** 1 Department of Gynaecology and Obstetrics, The Affiliated Hospital of Southwest Medical University, No. 25 Taiping Street, Jiangyang District, Luzhou, 646000 People’s Republic of China; 2 Sichuan Provincial Center for Gynaecology and Breast Diseases, The Affiliated Hospital of Southwest Medical University, Luzhou, 646000 China; 3 North Sichuan Medical College, Nanchong, 637000 China; 4 Department of Gynaecology, The Second People’s Hospital of Yibin, Yibin, 644000 China

**Keywords:** Hippo pathway, Breast cancer, Endocrine therapy, Endocrine resistance, Breast cancer stem cells, MicroRNA

## Abstract

Drug resistance is always a great obstacle in any endocrine therapy of breast cancer. Although the combination of endocrine therapy and targeted therapy has been shown to significantly improve prognosis, refractory endocrine resistance is still common. Dysregulation of the Hippo pathway is often related to the occurrence and the development of many tumors. Targeted therapies of this pathway have played important roles in the study of triple negative breast cancer (TNBC). Targeting the Hippo pathway in combination with chemotherapy or other targeted therapies has been shown to significantly improve specific antitumor effects and reduce cancer antidrug resistance. Further exploration has shown that the Hippo pathway is closely related to endocrine resistance, and it plays a “co-correlation point” role in numerous pathways involving endocrine resistance, including related pathways in breast cancer stem cells (BCSCs). Agents and miRNAs targeting the components of the Hippo pathway are expected to significantly enhance the sensitivity of breast cancer cells to endocrine therapy. This review initially explains the possible mechanism of the Hippo pathway in combating endocrine resistance, and it concludes by recommending endocrine therapy in combination with therapies targeting the Hippo pathway in the study of endocrine-resistant breast cancers.

## Introduction

The incidence of breast cancer ranks first among all female malignancies [1]. Most breast cancers are surely estrogen receptor-positive (ER +) and can depend on estrogen for tumor cell growth. The major treatment strategy for endocrine therapies focused on ER + breast cancer is estrogen deprivation. The current drugs to this end can be classed as aromatase inhibitors, selective estrogen receptor degraders, and selective estrogen receptor modulators. However, about one-third of patients still develop recurrence after long-term endocrine therapy [2]. Drug resistance to endocrine therapy has become a pivotal obstacle to treatment, although endocrine therapy is effective in reducing mortality and improving survival rates [3]. The reasons for drug resistance are multiple and complex, and they involve various molecules. Currently, targeted therapies combined with endocrine therapy have been shown to be effective for combating endocrine resistance, as illustrated in Table 1, which includes targets of Cyclin-dependent kinases (CDKs) 4 and 6, mammalian targets of rapamycin (mTOR) and phosphoinositide 3-kinase (PI3K). Their combination as first or second-line treatments for hormone receptor-positive metastatic breast cancer has been recommended by AMA guidelines, which was corroborated by a network meta-analysis [4]. Unfortunately, the combination of CDK 4 and 6 inhibitors still does not completely overcome drug resistance [5, 6]. The Hippo pathway, a newly targeted pathway, has been found to be related to multiple malignancies and modulation of this pathway may be able to effectively overcome endocrine resistance. In this review, the therapeutic potential of the Hippo pathway in the promotion of endocrine therapy is addressed, which provides theoretical references for the design of further studies and clinical consequences.

**Table 1:** Targeted agents approved for combination with endocrine therapy for the treatment of breast cancer

Targets	Agents	Mechanism	References
mTOR	Everolimus	Generates a complex that inhibits the activation of mTOR	[7]
CDK4/6	Palbociclib	Inhibits CDK4/6, allowing restoration of control of cell cycle	[8]
	Ribociclib		[9]
	Abemaciclib		[10]
PI3K	Alpelisib	Specifically inhibits PI3Kα	[11]

*CDK* cyclin-dependent kinase, *mTOR* mammalian target of rapamycin, *PI3K* phosphoinositide 3-kinase

## Mechanism of endocrine resistance in breast carcinoma

The complex reasons of endocrine resistance involves the estrogen receptor (ER) pathway, the growth factor receptor (GFR) pathways, and the CDK 4 and 6 pathway, as well as epigenetic modification. In recent years, the interdependence between BCSCs and drug resistance has emerged, and it often involves BCSC-related pathways, offering new insight into endocrine resistance.

### Refractory resistance facilitated by escaped BCSCs

BCSC-related markers, such as CD44^+^CD24^−/low^ [12], ALDH [13], CD133 [14],*Nanog* [15], *Sox2* [12], and *Sox9* [16], were found to be enriched or positive in tamoxifen-resistant [12–16], letrozole-treated [17], and fulvestrant-treated cells [18]. The proportion of cells bearing stem cell markers in resistant cell line stronger increased than that of non-resistant [12]. On the one hand, dormant and self-renewal deficient BCSC populations are generated during long-term endocrine therapy, leading to endocrine resistance after these cells exit from metabolic dormancy [19]. On the other hand, ER activity affects the enrichment of BCSCs via mutations in the *ESR1* gene (estrogen receptor-gene), including *Y537N*, *Y537S*, and *D598G*, which are involved in ligand-independent activation of ER [20]. Moreover, ER-α36 [21], ERβ [22], and G protein-coupled estrogen receptor (GPER) [23] are all involved in the promotion or maintenance of breast cancer stem cell character. Moreover, BCSC populations are considered to be estrogen receptor negative (ER −) and can regulate ER expression [24, 25], giving them the ability to generate ER + cells [26] or differentiate into cells that have lost ERα expression [27]. Therefore, the core reason for drug resistance is the stem cell behavior of breast cancers.

### The interaction between ER signaling, GFR signaling, and BCSC-related pathways

**Fig. 1:**
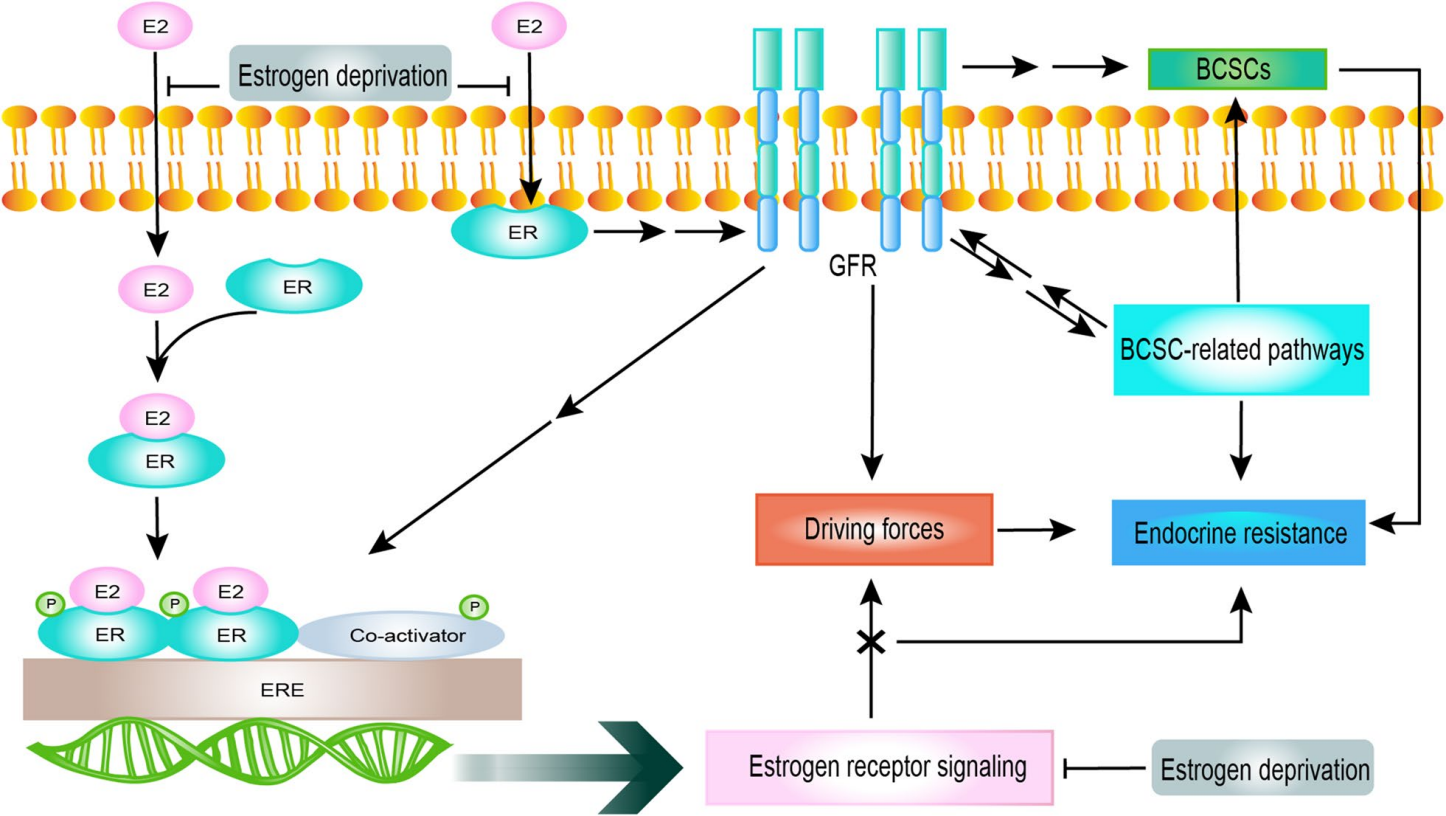
Illustration of preliminary mechanisms driving endocrine resistance in breast cancer. ER signaling drives cell growth in breast cancer. These driving forces convert from ER signaling to GFR signaling, with the interruption or the gradual reduction of ER signaling and the increase of GFR signaling. Interactions between GFR signaling and BCSC-related pathways will form stronger driving forces and lead to refractory breast cancer

BCSC-related pathways include the Hippo pathway, and those that signal via Hedgehog (Hh) signaling, transforming growth factor β (TGF-β) signaling, Notch signaling, PI3K/Akt/mTOR pathway, Wnt pathway, epidermal growth factor receptor (EGFR) pathway, ER signaling, and mitogen-activated protein kinase (MAPK) pathway. GFR signaling includes human epidermal growth factor receptor 2 (HER2) signaling, fibroblast growth factor receptor 1 (FGFR) signaling, and PI3K/Akt pathway, and they are three BCSC-related pathways. And other GFR signaling pathways are insulin-like growth factor 1 receptor (IGF-1R) signaling, MAPK signaling, and EGFR pathway. ER signaling is considered to be the main source of driving forces for the growth of ER + breast carcinomas. Changes in ER signaling are the first step for acquiring endocrine resistance, and this can include downregulation of ERα expression or loss of ERα expression caused by mutation or methylation of ERα-related genes [28]. These driving forces convert from ER signaling to GFR signaling [29, 30], as described in Fig. 1. The cross talk between GFR signaling and BCSC-related pathways may be the next step that leads to refractory resistance [28, 31, 32]. The preliminary relationship between part of BCSC-related pathways and endocrine resistance is diverse and complex, as illustrated in Table 2. And several signaling pathways of GFR are also BCSC-related pathways in this table. This reveals the dual roles of PI3K/Akt/mTOR, EGFR and MAPK pathways involve in endocrine resistance and breast cancer stem cell character. Moreover, it also reveals the extensive and complex role about BCSCs and BCSC-related pathways for endocrine resistance. BCSCs can depend on the GFR signaling pathway to survive, and escape from estrogen deprivation based on their ER − status [29, 33, 34]. Eventually, BCSCs will become the root cause of refractory resistance and escort breast cancers toward “permanent survival”. The combinations of targeted therapies focused on IGF-1R [35], HER2 [36, 37], or epidermal growth factor receptor [38–40] and endocrine therapy, even dual-targeting therapies plus endocrine therapy, have been shown not to reverse endocrine resistance or significantly enhance the effects of endocrine therapy. It has been suggested that breast cancers can still maintain endocrine resistance through the synergy of other pathways after inhibiting a small portion of these pathways. In conclusion, the process of endocrine resistance in breast carcinomas is the synergy of multiple mechanism. A new solution needs to be conceived.

**Table 2:** Preliminary mechanism of BCSC-related pathways associated with endocrine resistance

BCSC-related pathways	Association with BCSCs	Association with endocrine resistance
PI3K/Akt/mTOR	Heregulin-1β increases the cell fraction of CD44^+^/CD24^−^ and activates both MAPK and PI3K/Akt/mTOR pathways [41]. CD44^+^CD24^−^CD45^−^ BCSCs isolated from primary ERα-positive breast carcinoma exhibit hyperactive genes involved in the PI3K pathway, including *PIK3R1*, *PIK3R2*, *PIK3CA* and *EGFR *[42]. CSC population plays a vital role during endocrine resistance by activation of the mTOR pathway [43]	Heregulin-1β also can regulate ER/HER family expression by upregulating HER family mRNA expression and downregulating ERα mRNA expression [41]. Deactivation of PI3K/Akt/mTOR signaling increases the sensitivity of breast cancer cells to tamoxifen [44]. The activation of the PI3K/Akt/mTOR signaling in response to endocrine therapy may indeed result in acquired endocrine resistance [45]
EGFR	ER + breast cancer cells become resistant to tamoxifen treatment through enhanced regulation of the loops of ER-α36-EGFR/HER2 [46]. Rapid ER signaling mediated by ER-α36 is involved in regulation of BCSCs [47]. And ER-α36 could also positively regulates the population of ALDH1 breast cancers and HER2 expression [48]. EGFR overexpression regulates the CD24^low^CD44^high^ and ALDH^+^ in BCSC phenotypes as well as clonal formation [49]	EGFR expression and HER-2 amplification exert lower levels of ER and less response to tamoxifen [50]. Loss of ER by activation of EGFR pathway induces tamoxifen resistance and aberrant EGFR expression which is relative to a poor prognosis in ER + breast carcinomas [51]
MAPK	Laminin reduces the expression of *Nanog* and *Sox2*, and inhibits the ability to form secondary mammospheres [52]. The effects of laminin are mediated by MAPK/ERK pathway [52]. The inhibitor of MAPK/ERK pathway reduces the size of mammospheres in MCF7 cells [53]. High expression of phosphorylated p38γ MAPK is associated with CSC population in breast cancer cell lines [54]	The influences of CXCR4 overexpression are related with SDF-1–mediated stimulation of downstream signaling through p38 MAPK and ERK1/2 [55]. Overexpression of CXCR4 improves the expression of ER-mediated gene and alters endocrine therapy sensitivity [55]. Overexpression of Krüppel-like factor 4 inhibits p38 and ERK signaling, and results in increased sensitivity to tamoxifen[56]. Heregulin-1β-stimulated pathways enable ERα-positive breast cancer cells to everolimus resistance via MAPK pathway [41]
Wnt	Wnt/β-catenin pathway is activated, and exhibits stem-like features with upregulated cancer stem cell markers *Nanog* and ALDH1 in endocrine-resistant breast cancer [57]	Molecules of Wnt pathway may be regarded as an intrinsic factor in the transition to tamoxifen resistance [58]. Endocrine-resistant cells have enrichment of stem-like properties including increase of BCSC-related genes, stimulation of Wnt/β-catenin pathway, and formation of mammospheres [57].Sensitivity in MCF7 stem cells to tamoxifen is increased by inactivation of Wnt/β-catenin pathway [59]
Hh	The activated Hh pathway regulates BCSCs by increasing *GLI1* expression and enhancing the expression of *Sox2 *[60]	Treatment of tamoxifen-resistant xenografts with a Hh inhibitor suppresses tumor growth [61]. Hh pathways is regulated by PI3K pathway which has a protection of key components in Hh signaling from proteasomal degradation [61]. Hh signaling is activated by PI3K pathway in the endocrine-resistant breast tumor cells [62]. The levels of the Hh signaling component *GLI1* are significantly improved in tamoxifen-resistant MCF-7 cells and T47D cells [61]
Notch	CD133^hi^ BCSCs sustain self-renewal ability by an ER-independent manner, which exhibits the suppression of ER signaling caused by hormonal therapy and the upregulation of Notch3 [63].Short-term treatment with tamoxifen or fulvestrant inhibits cell proliferation but increases BCSC activity via activation of JAG1-NOTCH4 receptor both in xenograft tumors and patient-derived samples [18]	The reduction of Notch3 expression and activity abrogates hormonal therapy resistance and the expansion ability of CD133^hi^ cancer cells [63]. The activation of Notch4 results in poor response of MCF7 to tamoxifen [64]. In xenograft tumors with tamoxifen resistance, Notch4 suppression inhibits BCSC activity [18]
TGF-β	TGF-β induces sphere-forming efficiency in MDA361 and BT474 cells by a dose-dependent manner [65]. However, TGF-β inhibits sphere-forming efficiency in MCF7 cells [65]. Treatment with TGF-β increases ALDH^+^ and CD24^low^/CD44^+^ CSC-enriched population in breast cancer cell lines [66]	The plasma levels of TGF-β are upregulated in the group of these patients with endocrine-resistant breast cancer, as compare with the healthy group [67]. High PI3K and Hh pathway activity is relative to shorter PFS of metastases during tamoxifen treatment, and high TGF-β and PI3K pathway activity with worse response in treatment [68]. The secretion about active TGF-β can be induced to more than eightfold with the treatment of anti-estrogen in MCF-7 cells, and the active TGF-β secreted by the MCF-7 cell line also could suppress the growth of an ER − breast cancer cell line [69]

*PFS* progression-free survival

## The Hippo pathway

The Hippo pathway is also called the Salvador/Warts/Hippo pathway, and can precisely control the number of cells and stop organism growth in a timely manner during the development of mammals.

### Hippo pathway and its dysregulation

**Fig. 2:**
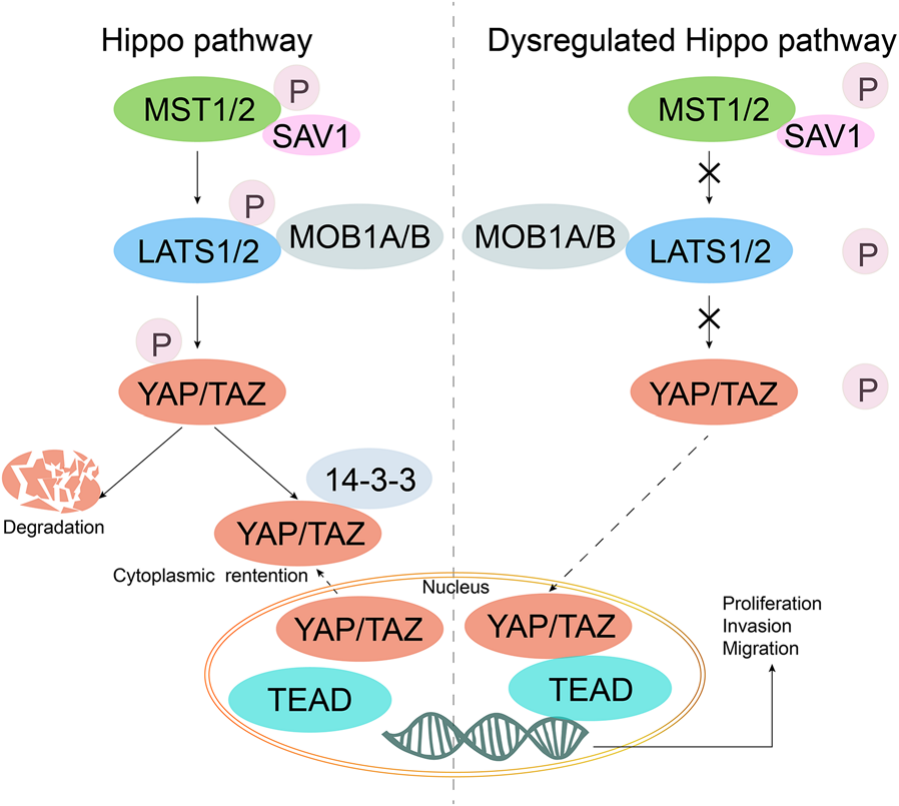
Illustration of the canonical Hippo pathway and its dysregulation. MST1/2 and LATS1/2 are kinases. SAV1 (WW45) and MOB1A/B are adaptors. The phosphorylation cascade in the Hippo pathway can negatively regulate YAP/TAZ and inhibit the proliferation-promoting effects of YAP/TAZ. Dysregulation leads to proliferation, invasion, and migration of malignancies via the nuclear translocation of YAP and TAZ

When the phosphorylation cascades of the Hippo pathway become blocked, these cells will differentiate abnormally and gradually develop into malignancies [70]. Then, inhibited or disabled Hippo phosphorylation cascades of Hippo pathway will further facilitate the invasion and migration of tumor cells [71, 72]. The mechanism of the canonical Hippo pathway and its dysregulation is described in Fig. 2. The activated Hippo pathway participates in the reasonable regulation of apoptosis and cell growth, when transcriptional coactivator with PDZ-binding motif (TAZ), and homologous component the yes-associated protein (YAP) are inactivated via their phosphorylation cascade. A variety of upstream signals activate mammalian sterile20-like (MST) kinases, and then MST kinases phosphorylate large tumor suppressor (LATS) kinases [73]. The activated LATS1/2 kinases can change the status of the phosphorylation and distribution of YAP/TAZ [74] resulting in the arrest of the cell cycle [75, 76]. LATS [77]and MST [73] are considered to have antitumor effects, and play negative roles in the regulation of TAZ and YAP. The entry of phosphorylated YAP/TAZ into the nucleus is restricted, and YAP/TAZ will be degraded after transferring into the cytoplasm from the nucleus, which can turn off antiapoptotic gene transcriptions and the cell cycle progression [78]. Conversely, when the Hippo pathway is dysregulated, the expression of downstream target genes of the Hippo pathway will promote cell proliferation and inhibit apoptosis genes, facilitating malignancies [79]. Dysregulation of the Hippo pathway showed results in excessive activation [80] or increased nuclear localization of YAP/TAZ by relatively decreasing YAP/TAZ phosphorylation [81], inhibiting LATS [82] and MST [83, 84]. Therefore, activated YAP and homologous TAZ are the core regulators of this pathway, since both of them exert oncogenic roles [85, 86] in the presence of a dysregulated Hippo pathway. Moreover, the low expression of MST and LATS will lead to the loss of control of YAP and TAZ [87]. Beyond this, additional non-canonical roles of the Hippo pathway in breast cancer have gradually emerged in recent years.

### Breast cancer stem cell character suppression by the Hippo pathway

It has been confirmed that TAZ, YAP, LATS, and TEA domain family members (TEAD) are involved in the regulation of BCSCs. Cordenonsi et al. [88] first linked the Hippo pathway to the concept of BCSC proliferation. Since then, studies have confirmed that BCSCs can be facilitated by TAZ [89–93]. YAP [94–97], as a homology of TAZ, the same as LATS [98] or TEAD [99], can also regulate BCSCs. Liu et al. [90] showed the restoration of sensitivity to tamoxifen and suppressed BCSCs by inhibiting TAZ expression. Moreover, dysregulation of the Hippo pathway is one of the prerequisites for the progress of an epithelial-mesenchymal transition (EMT) [100], which enables tumor to maintain the stem cell character [88]. MiR-520b activates the Hippo/YAP signaling pathways by targeting LATS, increasing the mRNA level of BCSC markers, such as CD133, CD44, and ALDH1, and the EMT marker N-cadherin in breast cancer [94]. Therefore, reactivating the Hippo pathway can effectively combat endocrine resistance and the expression of several oncogenes facilitated by BCSC transition.

## The cross talk between the Hippo pathway and multiple pathways involved in endocrine resistance

Breast cancer can rely on ER signaling to promote cell proliferation, which is different from other tumors. The Hippo pathway is indeed associated with almost all pathways related to endocrine resistance, including ER signaling. Researchers have found partial cross talk between the Hippo pathway and ER signaling. Recent studies have found that targeting and activating the Hippo pathway can negatively regulate BCSCs and overcome endocrine resistance. Moreover, it also seems to be involved in the integration of GFR signaling pathways and Hippo pathway. This indicates that the Hippo pathway may make an unexpected contribution to endocrine therapy in ER + breast cancers.

### The correlation between the Hippo pathway and the ER signaling pathway

**Fig. 3:**
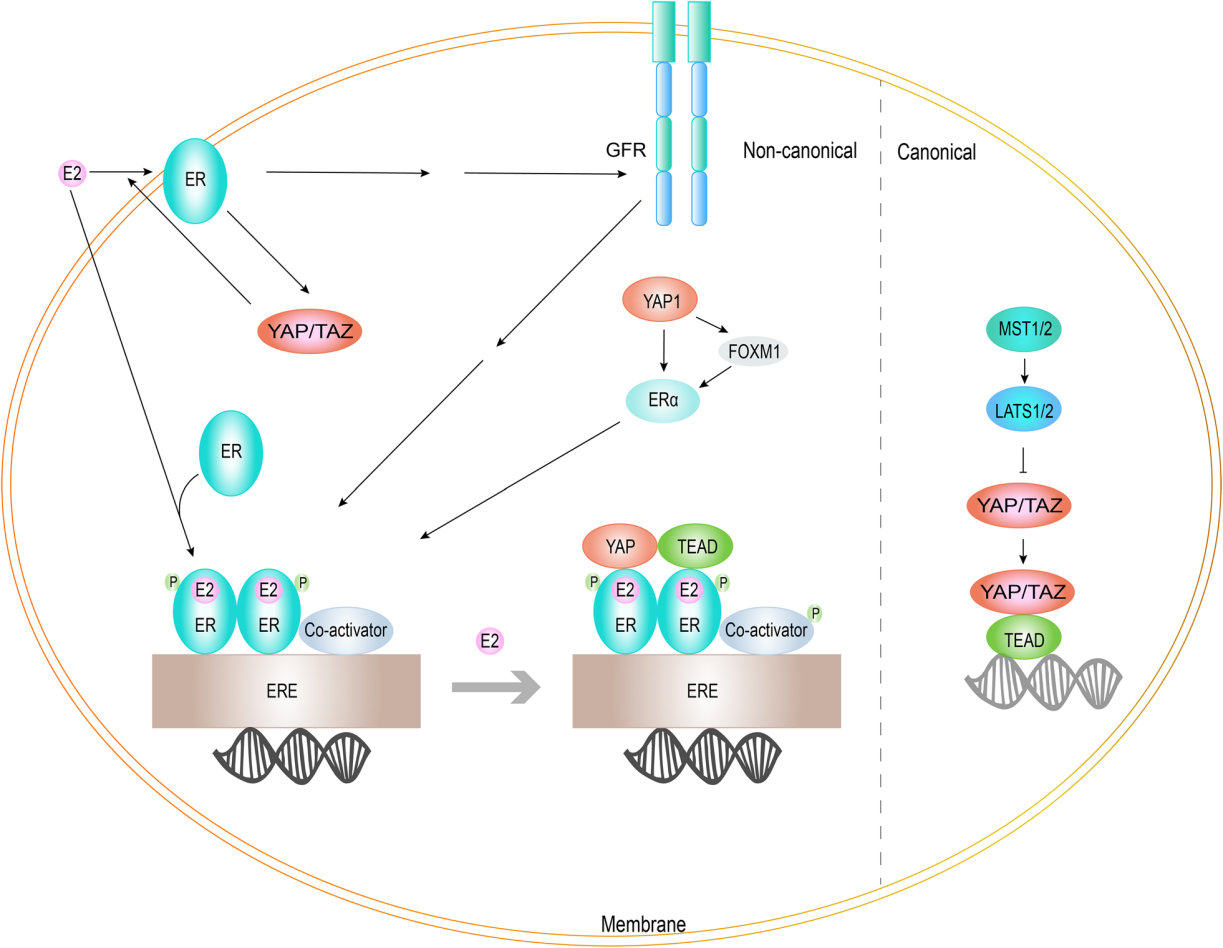
Preliminary illustration of non-canonical roles between the Hippo pathway and ER signaling in breast cancer. Feedback was formed between YAP/TAZ and ER. YAP and TEAD function as co-regulators of ER signaling to facilitate gene transcription of ER signaling genes

Preliminary non-canonical roles between the Hippo pathway and ER signaling in breast cancer is described in Fig. 3. On the one hand, the non-canonical roles of the components in Hippo pathway play a vital role in the management of ER signaling. YAP1 and TEAD4, co-regulators of ERα on enhancers, are augmented upon estrogen stimulation and transduction of target genes of ER signaling [101]. In addition, the expression of ERα was shown to directly be increased by YAP1 or indirectly mediated by the fork head box protein M1 (FOXM1) in the absence of the tumor suppressor Ras-association domain family 1 [102]. YAP/TAZ have also been shown to mediate the process of target gene induction by GPER [103]. On the other hand, ER can also affect the Hippo pathway. A study on mouse morula and trophoblast stem cells found that the nuclear localization of YAP was indeed regulated by ERα [104]. Moreover, the invasion and migration of TNBC were inhibited when the nuclear localization of YAP was inhibited, while sometimes the same situation did not appear in ER + breast cancer, which may have been related to the compensatory increase in the nuclear localization of YAP mediated by ER [105]. The status of ER can also affect the interaction between cellular retinoic acid binding protein 2 (CRABP2) and LATS to regulate the Hippo pathway and modulate sensitivity to endocrine therapy [106]. Moreover, GPER facilitates the progression of breast cancer by activating YAP/TAZ [103]. A study of tumor breast (226 samples) and normal (40 samples) from microarray samples found that the level of YAP expression was evidently downregulated in invasive cancer samples compared to normal tissues samples, and decreased expression of YAP was remarkably associated with ER − status [107]. This suggested that invasive breast cancer cells with reduced expression of YAP were more likely to be ER − and may have an lower threshold for becoming resistant to endocrine therapy. Feedback regulation of hormone receptors on the Hippo pathway in turn was weakened by the downregulation of ERα expression, and also was decreased by downward fluctuation of YAP. It may be the explanation for the lower YAP levels in invasive breast cancer are relative to normal breast tissue. The non-canonical Hippo pathway can in turn act on ER receptors to antagonize endocrine therapy, which can eventually leads to driving forces for tumor growth conversion to GFR signaling after estrogen-deprivation therapy. The regulation of the Hippo pathway for breast cancers dependent on different ER status and the different stage of endocrine resistance may be different [98, 106]. Thus, the study of the Hippo pathway in breast cancer cannot be generalized like other hormone-independent tumors.

### Antagonism of the Hippo pathway for endocrine resistance in ER + breast cancer

**Fig. 4:**
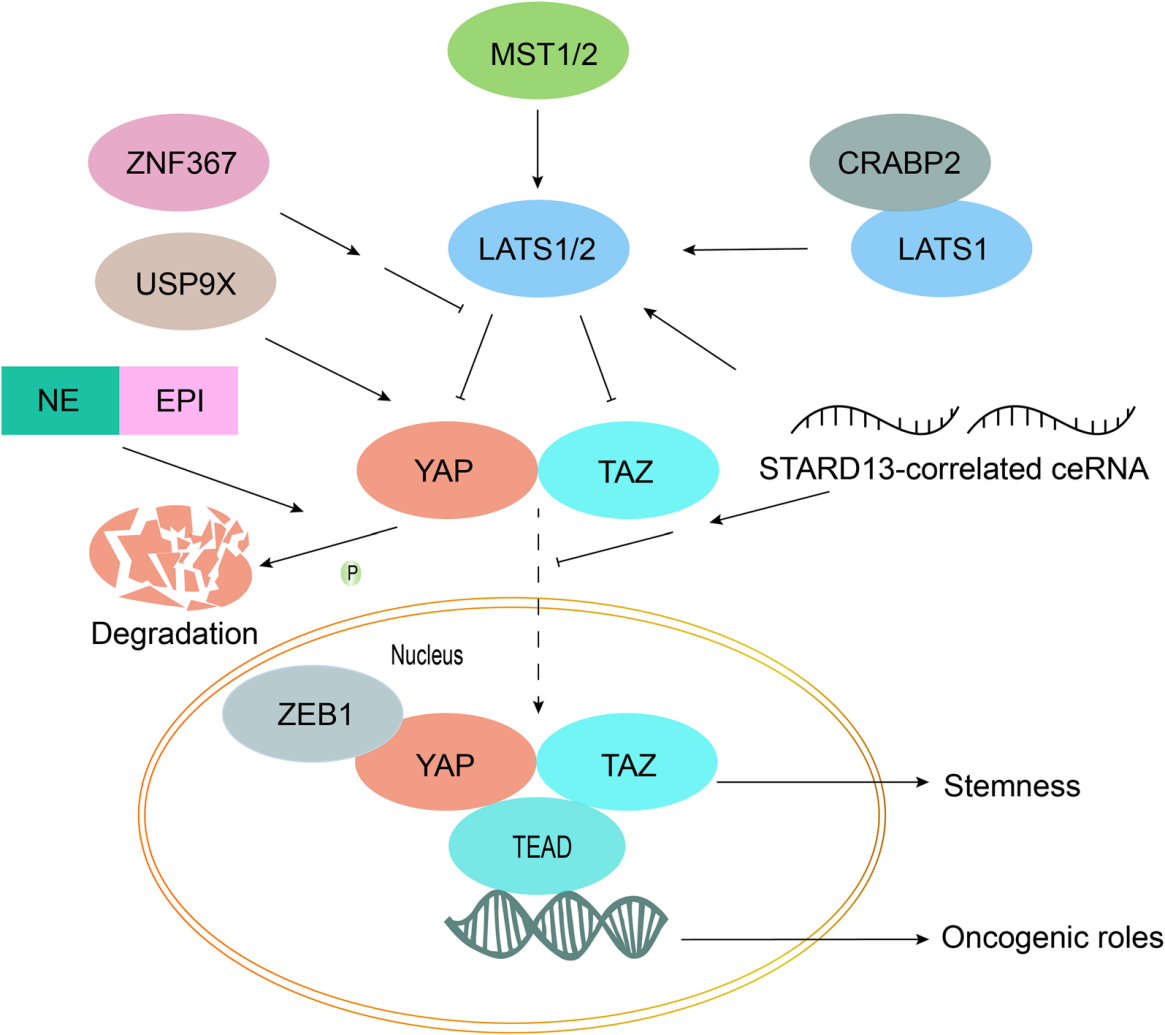
Illustration of the roles of the Hippo pathway in ER + breast cancer. Overexpression of ZNF367 facilitates metastasis and activates Hippo/YAP signaling by inhibiting LATS [110]. Overexpression of USP9X stimulates cell proliferation by deubiquitinating and stabilizing YAP1 [111]. NE and EPI suppress breast cancer via rapid phosphorylation and cytoplasmic retention of YAP [112]. ZEB1, a transcriptional activator, interacts with YAP1 and promotes transcription [113]. The interaction of LATS1 and CRABP2 inhibits the ubiquitination of LATS1 to suppress cell invasion [106]. The STARD13-correlated ceRNA network regulates TAZ distribution, and it can inhibit the stem cell character of breast cancer through upregulation of LATS1/2 [114]

Studies in the MCF7 cell line (ER-positive breast cancer) have confirmed that correcting dysregulation of the Hippo pathway is a feasible scheme to inhibit breast cancer and overcome acquired drug resistance. The roles of the Hippo pathway in ER + breast carcinoma are described in Fig. 4. In summary, YAP and TAZ surely are carcinogenic. Although the regulation of Hippo has been rarely studied in endocrine-resistant cells, the great prospect for modulation of the Hippo pathway has become an important research object. Zhou et al. [103] found that GPER’s role in inducing endocrine resistance could be regulated by the Hippo pathway and found that TAZ was overexpressed in GPER^hi^ breast cells. GPER activated YAP/TAZ, suggesting that blockage of GPER by knockdown of YAP/TAZ was a great strategy for overcoming tamoxifen resistance in GPER^hi^ breast cancers. Moreover, Zheng et al. [108] found that the YAP-glycolysis axis was also a target for overcoming tamoxifen resistance, based on the fact that the Hippo pathway was downstream of GPER. Li et al. [109] confirmed that downregulated YAP phosphorylation and upregulated YAP nuclear translocation directly resulted in tamoxifen resistance, which was reversed by YAP silencing.

### Cross talk between the Hippo pathway and BCSC-related pathways

**Fig. 5:**
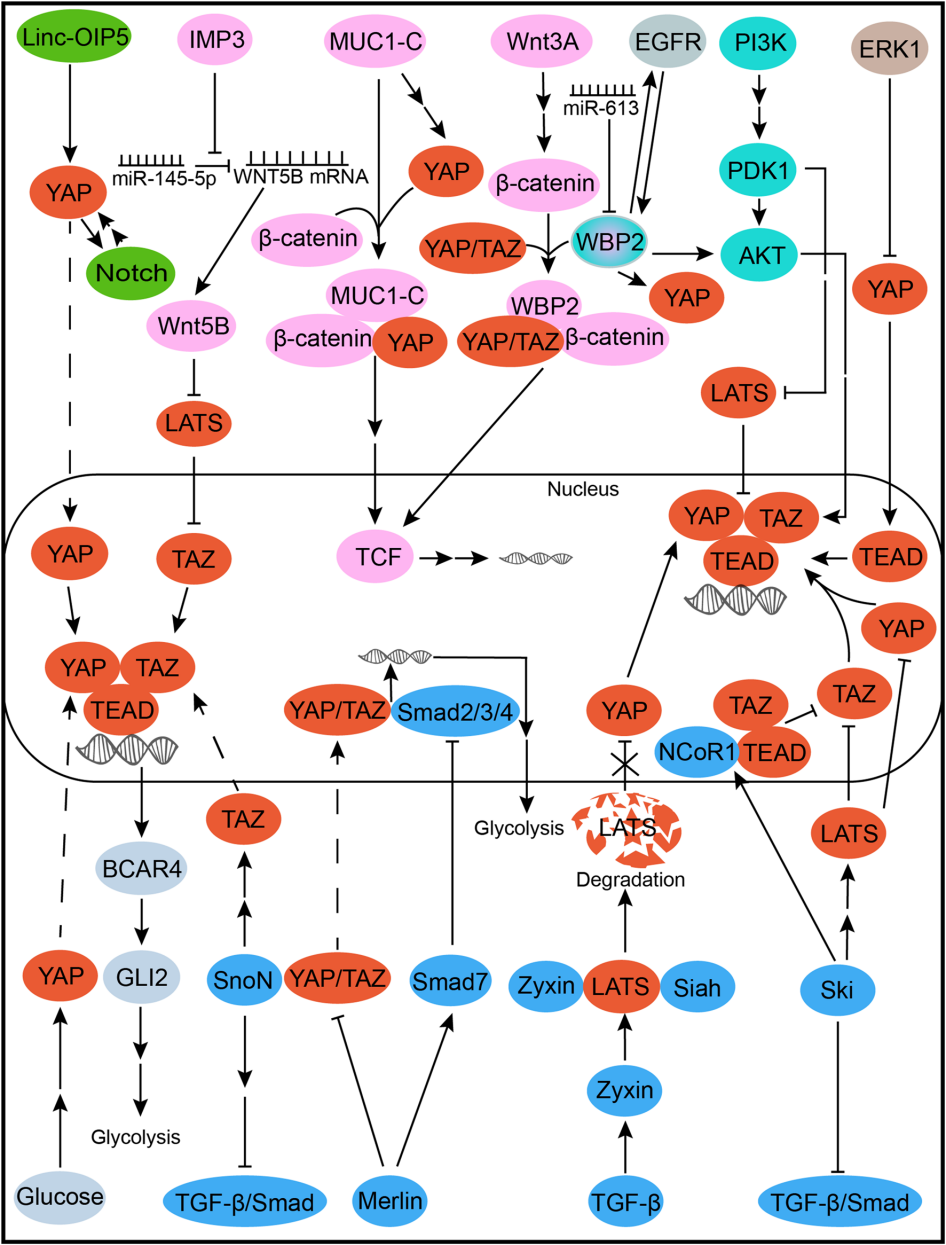
Illustration of the crosstalk of the Hippo pathway and BCSC-related pathways. Linc-OIP5 promotes transcription via forming a positive feedback circuit between YAP and Notch signaling [115]. IMP3 indirectly promotes Wnt5B via miR145-5p and facilitates TAZ-driven gene expression [116]. YAP and TAZ can promote the Wnt/β-catenin/TCF axis and induce target genes by interaction with β-catenin, while WBP2 integrates the Hippo, Wnt, and PI3K pathway [117–119]. Mir-613 inhibits EGFR via directly inhibiting WBP2 and positive correlation of EGFR and WBP2 is confirmed, while EGFR promotes WBP2 phosphorylation, contributing to integration of the Wnt/β-catenin and Hippo pathways [118, 119]. YAP/TAZ mediate the synergistic function and oncogene expression induced by the PI3K and dysregulated Hippo pathways [120]. The MAPK/ERK1 pathway negatively regulates breast cancer proliferation by inhibiting YAP/TEAD [121]. YAP induces gene transcription and promotes glycolysis by wiring up the Hh/GLI2 axis [108]. The SnoN oncoprotein exerts negative feedback regulation on TGF-β signaling, while promoting TAZ signaling and enhancing gene transcription in breast cancers [122]. Zyxin forms a ternary complex with LATS and Siah, which facilitates the degradation of LATS, activation of YAP and subsequently cell proliferation [123]. The tumor suppressor Merlin can inhibit YAZ/TAZ and maintain Smad7 stability, suppressing the adaptive glycolysis facilitated by the interaction between YAP/TAZ and Smads [124]. Ski inhibits breast cancer by suppressing TAZ in a LATS-dependent manner or in a LATS-independent manner, in which NCoR1 is recruited by Ski and suppresses TAZ by binding to the TEAD-TAZ complex [125]

The Hippo pathway plays a “co-correlation point” role in several networks of BCSC-related pathways, including Notch, Wnt, EGFR, PI3K/Akt, MAPK/ERK1, Hh/GLI2,TGF-β pathway. Clara et al. [85] first proposed that the Hippo pathway may be the “hub” of cancer stem cell related pathways. The cross talk between Hippo and BCSC-related pathways except ER signaling has been shown as described in Fig. 5. Hippo pathway does crosstalk these seven pathways, and there forms “bridges” between Hippo, Wnt, EGFR and PI3K/Akt pathways through WBP2. And these “bridges” do enable GFR signaling pathways and BCSC-related pathways to form crosstalk. Extensive integration and local interlinkages reveal the advantages of Hippo pathway in regulation of drug resistance through breast cancer stem cell character. In addition, Hippo, TGF-β, Hh, MAPK, Wnt, PI3K/Akt, Notch, EGFR, and ER signaling were all associated with the EMT process. It has been further revealed that these pathways facilitate the synergistic regulation of breast cancer and can cause endocrine resistance in breast cancer. It is of no doubt, then, that the dysregulation of the Hippo pathway indeed facilitated the progression of breast cancer, and exert intricate cross talk on BCSC-related pathways. BCSCs are the key to maintaining refractory survival and drug resistance for tumor cells, which is based on the coregulation of these pathways. Therefore, the “co-correlation point” role of the Hippo pathway in BCSC-related pathways may highlight a new solution for overcoming endocrine resistance.

### The interrelation between the Hippo pathway, GFR, and the Cyclin-dependent kinase 4 and 6 pathways

The rest of GFR signaling pathways and Cyclin-dependent kinase 4 and 6 pathways involved in endocrine resistance also exert cross talk on the Hippo pathway. With regards to patients with luminal B subtypes, samples with low TAZ resulted in higher pathological complete response rates after trastuzumab-based neoadjuvant therapy, suggesting that HER2 linked with TAZ expression in a consistent manner [126]. YAP/TAZ dephosphorylation and overexpression increased in trastuzumab-resistant breast cancer cells, suggesting that the dysregulated Hippo pathway further facilitated cancer cells in coordination with HER2 [127]. Inhibiting YAP and TAZ could eliminate Lapatinib resistance, suggesting that dual target therapy for HER2 and the Hippo pathway had good prospects [128]. The Hippo pathway mediated FGFR signaling, the MAPK pathway, and PI3K signaling during tumorigenesis, and YAP/TAZ were shown to be possible therapeutic targets in RTK-driven cancers [129]. The phosphorylation of MST1 depended on the activity of fibroblast growth factor receptor 4 kinase. Moreover, short-term suppression or knockdown of FGFR4 led to increased activation of MST1/2 [130]. The IGF-1R/ YAP axis has been shown to be involved in the growth of TNBC [131]. Dysregulation of the Hippo pathway also can increase resistance to CDK4/6 inhibitors through accumulation of TAZ and YAP transcription factors on the promoter of Cyclin-dependent kinase 6 [5]. These studies further demonstrated the great potential of the Hippo pathway for remedying breast cancer.

### Integration of Hippo pathway in complex mechanism of endocrine resistance

**Fig. 6:**
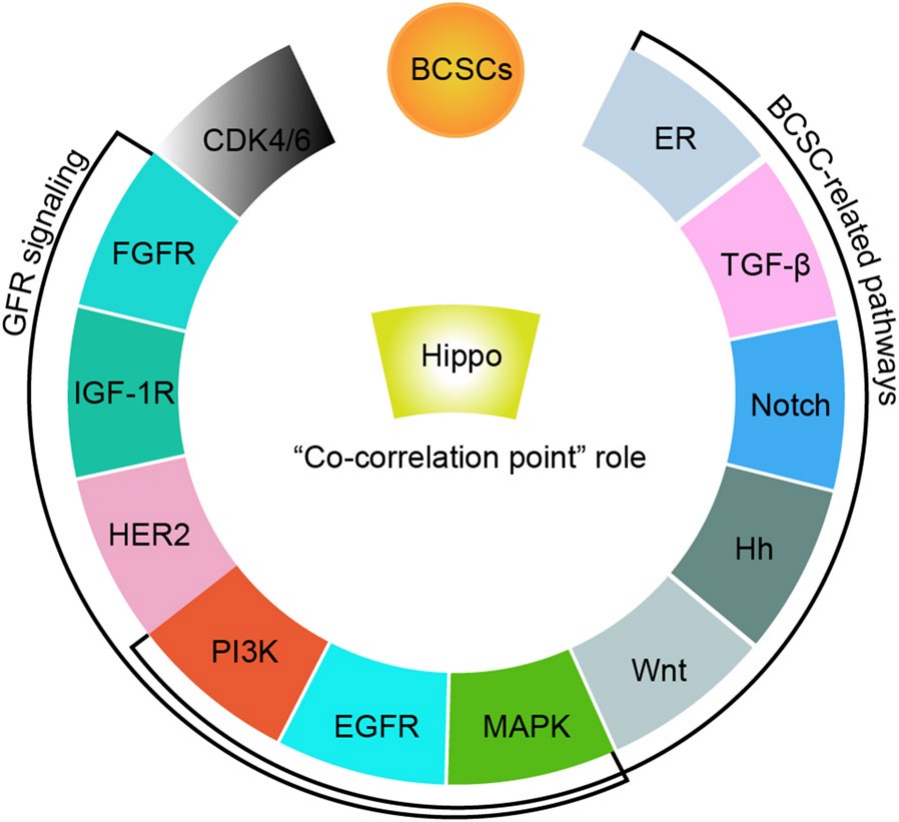
Illustration of the “co-correlation point” role of Hippo pathway in the multiple mechanism of endocrine resistance

These roles of Hippo pathway in the integration of ER signaling are unique to breast cancers. The regulation of Hippo pathway on cancer stem cells is also affirmed, including BCSCs. Hippo pathway can not only combat BCSCs, but also can integrate these multiple pathways involve in endocrine resistance, including GFR (PI3K/Akt/mTOR, EGFR, MAPK, HER2, IGF-1R and FGFR),BCSC-related pathways (Wnt, PI3K/Akt/mTOR, Hh, EGFR, Notch, MAPK, TGF-β, ER), and CDK4/6 pathway. The “co-correlation point” role of Hippo pathway in the multiple mechanism of endocrine resistance as described in Fig. 6. To sum up, utilizing the Hippo pathway as a therapeutic target for combating endocrine-resistant breast cancer may be a promising approach.

## Agents and miRNAs for research proposals focusing on the Hippo pathway

**Fig. 7:**
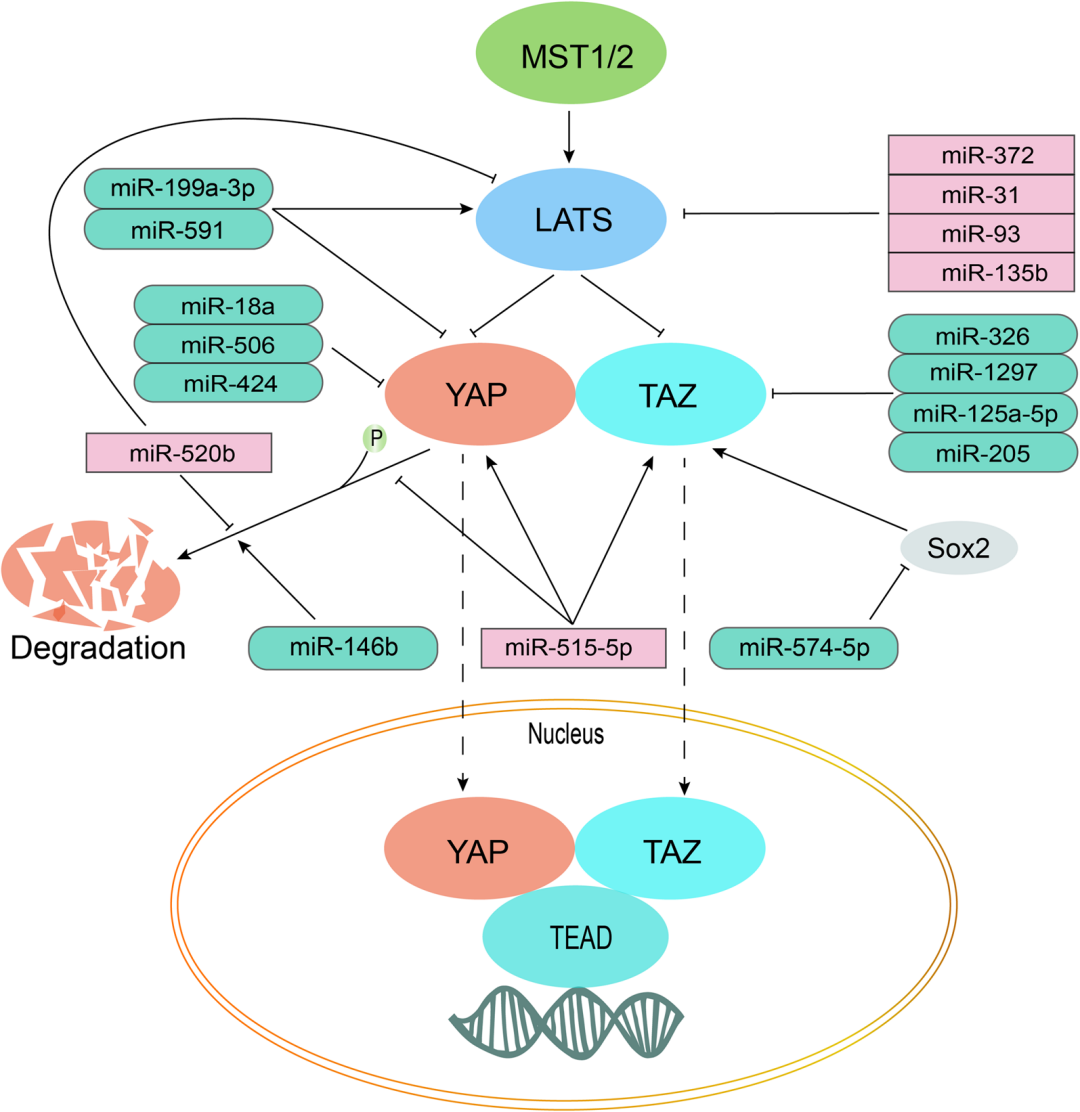
Illustration of miRNAs in regulation of the Hippo pathway. MiRNAs dysregulate the Hippo pathway by inhibiting LATS and phosphorylation of YAP, or directly upregulating YAP and TAZ. MiR-520b, miR-372, miR-31, miR-93, miR-135b and miR-515-5p are tumor promotors with the dysregulation of the Hippo pathway. On the contrary, miR-199a-3p, miR-591, miR-18a, miR-506, miR-424, miR-146b, miR-574-5p, miR-205, miR-125a-5p, miR-1297 and miR-326 are tumor suppressors with the activation of the Hippo pathways

In the process of endocrine therapy, the main driving forces for tumor growth were related to GFR signaling and even BCSC-related pathways. A dysregulated Hippo pathway did exert cross talk on these endocrine resistance-related pathways. A new therapeutic scheme that could be proposed to combat the “co-correlation point” of these pathways would focus on targeting the Hippo pathway. The combination of endocrine therapy and targeted therapy focusing on the Hippo pathway is expected to significantly improve the prognosis of patients with ER + breast cancers. There are many agents that can target the Hippo pathway, including Pevonedistat, Verteporfin, stains, and Metformin [85, 132]. Statins and Metformin are commonly used to regulate metabolism of lipids and glucoses. In recent years, there are clinical trials targeting the Hippo pathway by them, although no available results. Clinical trials are designed for targeting the Hippo pathway as illustrated in Table 3. Clinical trials of combination of endocrine therapy and agents targeting Hippo pathway for reference as illustrated in Table 4. Moreover, the regulation of miRNAs is also an alternative tool. MiRNAs are related to the Hippo pathway in breast cancers, as illustrated in Table 5 and Fig. 7. Partial miRNAs can also be involved in the regulation of EMT [94, 133–135] and the maintenance of stem cell character in breast cancer [94, 134, 135]. MiR-125a-5p [136], the miR-200 family [137], miR-375 [138], and miR-181b [139], have all been recommended as therapeutic agents, since all of them can regulate endocrine resistance and were confirmed to be related to the Hippo pathway in other tumors. Upregulation of miR-125a-5p in tamoxifen resistant MCF7 cells may inhibit the growth of BCSCs by suppression of TAZ, which is an effective promoter of BCSCs. It is expected that the results of preclinical and clinical data will confirm its role in the future.

**Table 3:** Designed clinical trials of agents targeting the Hippo pathway

Agents	Mode of action	Study phase	Outcome	Study Title	NCT number
Zoledronate, Atorvastatin	Inhibition of YAP/TAZ	Phase 2	No results available	Neoadjuvant Zoledronate and Atorvastatin in TNBC	NCT03358017
Atorvastatin	Inhibits TAZ	Phase 2	No results available	Targeting the Hippo transducer TAZ in breast cancer with Statins	NCT 02416427
Zoledronate	Inhibits YAP/TAZ	Phase 2	No results available	Pre-operative zoledronate in TNBC	NCT 02347163

**Table 4:** Clinical trials of endocrine therapy combined with agents targeting Hippo pathway for reference

Agents		Study phase	Outcome	NCT number	Possible mechanisms
Hippo pathway	Endocrine therapy				
Atorvastatin	Letrozole, Fulvestrant	Phase 2	No results available	NCT02958852	Stains inhibits YAP/TAZ nuclear localization, and suppressed the self-renewal capability of cancer stem cells via opposing nuclear TAZ activity [140] Metformin inhibits YAP nuclear localization [141]
Metformin, Simvastatin	Fulvestrant	Phase 2	No results available	NCT03192293	
Metformin	Toremifene	Phase 2	No results available	NCT02506790	
Metformin	Fulvestrant	Phase 2	No results available	NCT04300790	
Metformin	Everolimus, Exemestane	Phase 2	The clinical benefit rate was 54.5%	NCT01627067

**Table 5:** MiRNAs that regulate the Hippo pathway in breast cancer

MiRNAs		Tumor suppressor (−)/tumor promotor (+)	Cell lines	Targets	Mechanisms of regulation
MiR-326 [142]		–	MCF-7, MDA-MB-468	TAZ	Circular RNA 0000511 can eliminate the anti-tumor effect of miR-326 by upregulating TAZ
MiR-146b [143]		–	MCF-7	p-YAP	The process of MUC19 reducing YAP phosphorylation is inhibited by miR-146b
MiR-199a-3p [133]		–	MDA-MB-231	LATS1, YAP1	miR-199a-3p suppresses YAP1 and upregulates LATS1
MiR-574-5p [135]		–	MDA-MB-231, T47D	TAZ	miR-574-5p targets *Sox2* to suppress TAZ
MiR-1297 [144]	–		MDA-MB-231, MDA-MB-468	TAZ	miR-1297 inhibits TAZ
MiR-125a-5p [145, 146]	–		MDA-MB-468, BT549, tamoxifen resistant MCF7	TAZ	miR-125a-5p directly inhibits TAZ expression. Downregulation of *CYTOR* decreases protein and mRNA levels of TAZ in tamoxifen resistant MCF7 cells, which is rescued by miR-125a-5p suppression
MiR-515-5p [147]	+		MDA-MB-231, MDA-MB-453	YAP, TAZ, p-TAZ	Knockdown LINC00673 reduces the level of YAP/TAZ and increases p-YAP through miR-515-5p inactivation
MiR-591 [148]	–		MCF-7, SKBR3	YAP, LATS	miR-591 inhibits YAP and upregulates LATS
MiR-520b [94]	+		MCF-7, MDA-MB-231	LATS2, p-YAP, YAP	miR-520b promotes migration activity and stemness of breast cancer, which can be abolished by overexpression of LATS2. miR-520b upregulates nuclear YAP and inhibits LATS2 as well as p-YAP
MiR-372 [149]	+		MCF-7, MDA-MB-231	LATS2	miR-372 inhibits LATS2
MiR-18a [150]	−		Trastuzumab-resistant SKBR-3	YAP1	miR-18a directly inhibits YAP1
MiR-424 [151]	−		MDA-MB-231, HCC-1937	YAP	miR-424 inhibits YAP
MiR-205 [134]	−		SUM159	TAZ	miR-205 inhibits TAZ, which is involved in the mammospheres formation and BCSC renewal
MiR-135b [152]	+		MDA-MB-231, MCF-7, 293 T	LATS2,	miR-135b inhibits LATS2
MiR-506 [153]	–		MDA-MB-231	YAP	miR-506 inhibits YAP
MiR-31 [154]	+		MDA-MB-231	LATS2	miR-31 inhibits LATS2
MiR-93 [155]	+		MT-1	LATS2	miR-93 inhibits LATS2

## Conclusion and prospects

Targeting the Hippo pathway has been researched in a variety of tumors and has been shown to have remarkable results. The combination of targeted therapy for the Hippo pathway and chemotherapy or other targeted therapies has also achieved initial results in breast cancers. Although its dysregulation can promote the occurrence and progression of tumors, reasonable regulation of this pathway can effectively inhibit tumors and combat endocrine resistance. However, the Hippo pathway is powerful, diverse, and complex. Breast cancers differ from other tumors, in that the former is derived from more of the non-canonical roles of the Hippo pathway via hormone receptors. More studies are needed to verify the feasibility and risk of regulation of the Hippo pathway in endocrine-resistant breast cancer. Moreover, our laboratory has shown that aromatase inhibitors such as Formestane can rely on ER-independent but androgen receptor-dependent roles to suppress ER + breast cancer, suggesting that aromatase inhibitors may be highly recommended as promising agents combined with targeting the Hippo pathway to significantly overcome endocrine resistance stimulated by estrogen-deprivation therapy in postmenopausal women [157]. Targeting the Hippo pathway will create promising new tools in the fight against endocrine-resistant breast cancer.

## Data Availability

Not applicable.

## Abbreviations

TNBC: Triple negative breast cancer
FOXM1: Fork head box protein M1
BCSCs: Breast cancer stem cells
USP9X: Ubiquitin-specific protease 9X
ER +: Estrogen receptor-positive
CDKs: Cyclin-dependent kinases
mTOR: Mammalian target of rapamycin
WBP2: WW domain-binding protein 2
E2: Estradiol
PI3K: Phosphoinositide 3-kinase
ER: Estrogen receptor
GFR: Growth factor receptor
PFS: Progression-free survival
GPER: G protein-coupled estrogen receptor
ER −: Estrogen receptor negative
Hh: Hedgehog
TGF-β: Transforming growth factor β
IMP3: Insulin-like growth factor-2 mRNA-binding protein 3
EGFR: Epidermal growth factor receptor
ERE: Estrogen response element
MAPK: Mitogen-activated protein kinase
CRABP2: Cellular retinoic acid binding protein 2
HER2: Human epidermal growth factor receptor 2
FGFR: Fibroblast growth factor receptor 1
NCoR1: Nuclear transcriptional corepressor N-CoR1
BCAR4: Breast cancer anti-estrogen resistance 4
IGF-1R: Insulin-like growth factor 1 receptor
YAP: Yes-associated protein
Linc-OIP5: Linc-Opa interacting protein 5
TAZ: Transcriptional coactivator with PDZ-binding motif
MUC1-C: The mucin 1 C-terminal subunit
MST: Mammalian sterile20-like
LATS: Large tumor suppressor
TEAD: TEA domain family members
ZNF367: Zinc finger protein 367
EPI: Epinephrine
NE: Norepinephrine
ceRNA: Competing endogenous RNA
ERK1: Extracellular signal-related kinases 1
